# The mobile gene cassette carrying tetracycline resistance genes in *Aeromonas veronii* strain Ah5S-24 isolated from catfish pond sediments shows similarity with a cassette found in other environmental and foodborne bacteria

**DOI:** 10.3389/fmicb.2023.1112941

**Published:** 2023-03-15

**Authors:** Saurabh Dubey, Eirill Ager-Wiick, Bo Peng, Angelo DePaola, Henning Sørum, Hetron Mweemba Munang’andu

**Affiliations:** ^1^Section for Experimental Biomedicine, Department of Production Animal Clinical Sciences, Faculty of Veterinary Medicine, Norwegian University of Life Sciences, Ås, Norway; ^2^State Key Laboratory of Biocontrol, Guangdong Key Laboratory of Pharmaceutical Functional Genes, School of Life Sciences, Southern Marine Science and Engineering Guangdong Laboratory (Zhuhai), Higher Education Mega Center, Sun Yat-sen University, Guangzhou, China; ^3^Angelo DePaola Consulting LLC, Coden, AL, United States; ^4^Department of Paraclinical Sciences, Faculty of Veterinary Medicine, Norwegian University of Life Sciences, Ås, Norway; ^5^Faculty of Biosciences and Aquaculture, Nord University, Bodø, Norway

**Keywords:** *Aeromonas veronii*, antimicrobial resistance, mobile gene cassette, virulence, tetracycline, environment, foodborne

## Abstract

*Aeromonas veronii* is a Gram-negative bacterium ubiquitously found in aquatic environments. It is a foodborne pathogen that causes diarrhea in humans and hemorrhagic septicemia in fish. In the present study, we used whole-genome sequencing (WGS) to evaluate the presence of antimicrobial resistance (AMR) and virulence genes found in *A. veronii* Ah5S-24 isolated from catfish pond sediments in South-East, United States. We found *cphA4*, *dfrA3*, *mcr-7.1*, *valF*, *bla*_FOX-7_, and *bla*_OXA-12_ resistance genes encoded in the chromosome of *A. veronii* Ah5S-24. We also found the tetracycline *tet(E)* and *tetR* genes placed next to the IS*5/*IS*1182* transposase, integrase, and hypothetical proteins that formed as a genetic structure or transposon designated as IS*5/*IS*1182/hp/tet(E)/tetR/hp*. BLAST analysis showed that a similar mobile gene cassette (MGC) existed in chromosomes of other bacteria species such as *Vibrio parahaemolyticus* isolated from retail fish at markets, *Aeromonas caviae* from human stool and *Aeromonas media* from a sewage bioreactor. In addition, the IS*5/*IS*1182/hp/tet(E)/tetR/hp* cassette was also found in the plasmid of *Vibrio alginolyticus* isolated from shrimp. As for virulence genes, we found the tap type IV pili (*tapA* and *tapY*), polar flagellae (*flgA* and *flgN*), lateral flagellae (*ifgA* and *IfgL*), and fimbriae (*pefC* and *pefD*) genes responsible for motility and adherence. We also found the hemolysin genes (*hylII*, *hylA,* and *TSH*), *aerA* toxin, biofilm formation, and quorum sensing (*LuxS*, *mshA,* and *mshQ*) genes. However, there were no MGCs encoding virulence genes found in *A. veronii* AhS5-24. Thus, our findings show that MGCs could play a vital role in the spread of AMR genes between chromosomes and plasmids among bacteria in aquatic environments. Overall, our findings are suggesting that MGCs encoding AMR genes could play a vital role in the spread of resistance acquired from high usage of antimicrobials in aquaculture to animals and humans.

## 1. Introduction

*Aeromonas veronii* is a Gram-negative bacterium ubiquitously found in different aquatic environments. It was first reported by Hickman-Brenner et al. (1987) as a new species in 1983. It is pathogenic to several fish species that include the top farmed species such as common carp (*Cyprinus carpio*), channel catfish (*Ictalurus punctatus*), tilapia (*Oreochromis niloticus*), and pangasius (*Pangasius hypophthalmus*) (González-Serrano et al., 2002; Smyrli et al., 2017, 2019; Wang et al., 2022). It causes hemorrhagic septicemia and skin ulcers in fish (Hoai et al., 2019; Tekedar et al., 2020) and diarrhea in humans (Roberts et al., 2006). Strain variations have been linked to virulence leading to studies aimed at identifying the virulence factors associated with mortalities (González-Serrano et al., 2002; Smyrli et al., 2017, 2019; Wang et al., 2022). The high mortalities experienced in aquaculture have led to use of antibiotics, thereby contributing to increase of antimicrobial resistance (AMR) (Roberts et al., 2006). As mentioned in our previous studies (Dubey et al., 2022a,b), the major limitation with most studies aimed at identifying AMR genes in bacteria is that they are mostly done by PCR that only detects AMR genes based on the primers used in the assay. This poses the risk of omitting important AMR genes whose primers are not included in PCR assays. Besides, PCR-based assays do not determine whether the AMR genes are intrinsically encoded in the chromosomes or extrinsically in plasmids. So, the use of whole-genome sequencing (WGS) able to detect all genes and their location in bacteria genomes is a better approach for elucidating the role of different bacteria species in the spread of AMR and virulence genes than PCR-based assays.

The spreading of AMR genes by horizontal transfer is contributing to involvement of bacteria species outside the 12 bacteria families enlisted to pose the greatest AMR threat to human health by the World Health Organization (WHO) (Willyard, 2017). As pointed out by White et al. (2001), the spread of AMR genes is enhanced when they form part of mobile gene cassettes (MGCs) or transposons. The MGCs were first identified as integrated AMR genes found in integrons in the early 1980s (Ward and Grinsted, 1982; Meyer et al., 1983; White et al., 2001). Although studies done this far have focused on cassettes carrying AMR genes, it is likely that the packaging in cassettes includes other genes such as virulence factors. As stated by White et al. (2001), MGCs facilitate horizontal gene transfer using various mechanisms that include mobilization of individual cassettes by integrons (Collis and Hall, 1992), movement of integrons having cassettes by transposases (Brown et al., 1996; Craig, 1996; Minakhina et al., 1999), dissemination of larger transposons carrying integrases (Liebert et al., 1999), and translocation of conjugative plasmids having integrases among bacteria (White et al., 2001). It is likely that most of the AMR genes associated with infections in aquaculture, livestock and humans are part of MGCs (Recchia and Hall, 1995). Yet, gene cassettes conferring resistance to antibiotics used in aquaculture have not been widely investigated as done in mammalian studies. Hence, it is unknown whether the AMR genes selected against drugs like tetracycline, sulphonamide, and trimethoprim widely used in aquaculture are packaged in MGCs. Thus, although previous studies have focused on identifying individual genes associated with resistance, the cassettes responsible for the spread of AMR genes has not been widely investigated for bacteria found in aquaculture.

In the present study we used WGS to profile all AMR and virulence genes found in *A. veronii* Ah5S-24 isolated from pond sediment obtained from the South East, USA by DePaola et al. (1988). Although in the previous study, they detected presence of Oxytetracycline-resistance (OTc^r^) and tetracycline-resistance (Tc^r^) by selecting for isolates that replicated on MacConkey agar containing oxytetracycline or tetracycline antibiotics, they did not determine whether the resistance gene was located in the chromosome or plasmids. Even though they showed the transfer of OTc^r^ and Tc^r^ resistance from the *Aeromonas* isolate to *Escherichia coli*, they did not determine whether the transfer was plasmid mediated or MGC. Thus, we wanted to determine whether the OTc^r^ and Tc^r^ resistance in the isolate was encoded in the chromosome or plasmid. We also wanted to determine whether the resistance detected was associated with a tetracycline genetic structure similar to that found in other bacteria species. We anticipate that data presented herein will underscore the importance of screening for MGCs carrying AMR genes from aquatic organisms with potential transmission to animals and humans.

## 2. Methodology

### 2.1. Bacteria culture, characterization, and antibiotic diffusion test

A suspected *Aeromonas hydrophila* isolated from pond sediments in the South-Eastern USA by DePaola et al. (1988) in 1988 was retrieved from the –80°C freezer at the Norwegian University of Life Sciences (NMBU), Ås, Norway. The isolate was kindly provided by Dr. Angelo DePaola, Gulf Coast Seafood Laboratory, United States. After thawing, the bacteria isolate was streaked on blood agar and incubated at 10°C for 5–7 days. Single colonies were streaked on tryptone soy agar (TSA) for purification followed by characterization using the Matrix-assisted laser Desorption/Ionization-Time of Flight (MALDI-TOF) mass spectrometry while DNA was extracted based on manufacturer’s protocol (Qiagen, Germany). Identification of the bacteria species was done by PCR using universal 16S rRNA primers 27F and 1492R. Phenotypic characterization of antibiotic resistance was done using the Kirby-Bauer disk diffusion test (Joseph et al., 2011). The commercial antibiotic discs (Neo-Sensitabs™, Rosco) used consisted of Penicillin (PEN-10 μg), Amoxicillin (AMOXY-30 μg), Ampicillin (AMP-10 μg), Ciprofloxacin (CIPR-5 μg), Cefoxitin (CFO-30 μg), Cephalothin (CEP-30 μg), Tetracycline (TET-30 μg), Gentamycin (GEN-10 μg), Rifampicin (RIF-5 μg), Sulfonamide (SULFA-240 μg), Trimethoprim (TRIM-5 μg), Erythromycin (Ery-15 μg), Nitrofurantoin (NI-300 μg), and (Colistin-CO-150 μg) (Table 1). A volume of 100 μl containing freshly cultured bacteria diluted at McFarland concentration of 10^8^ CFU/ml was spread on Müller Hinton agar followed by putting the antibiotic discs on the bacteria lawn. Next, the plates were incubated at 30°C overnight followed by measuring the susceptibility or resistance based on the Clinical and Laboratory Standards Institute (CLSI) guidelines (Kahlmeter et al., 2006; Cockerill et al., 2012).

**Table 1:** Overview of antibiotic resistence genes detected in the draft genome of *Aeromonas veronii* AhS5-24 together with phenotypic antibiotic susceptibility testing results using disk diffusion assay.

Resistance mechanism	Resistance gene	Antibiotic class	Antibiotic	Results
Antibiotic inactivation	*bla*_FOX-7_	Cephamycin	Cefoxitin (CFO30)	R
	*bla*_OXA-12_	Cephalosporin	Cephalothin (CEP 30)	R
	*cphA4*	*β*-lactams	Amoxicillin (AMOXY)	R
Antibiotic efflux	*tet(E)*	Tetracycline	Tetracycline (TET30)	R
	*MexB*	Sulfonamide, *β*-lactams	Sulfonamide (SULFA)	I
	*CRP*	Macrolide	Erythromycin (ERY15)	S
Antibiotic target alteration	*mcr*-7.1	Peptide	Colistin (CO150)	S
	*vatF*	–	–	–
Antibiotic target replacement	*dfrA3*	Diaminopyrimidine	Trimethoprim (TRIM5)	I
Other resistance mechanism		Fluoroquinolone	Ciprofloxin (CIPR5)	S
		Aminoglycoside	Gentamicin (GEN10)	S
		Nitrofuran	Nitrofurantoin (NI300)	S
		Rifamycin	Rifampicin RIF.5	S

### 2.2. DNA extraction

Genomic DNA (gDNA) was extracted as previously described (Becker et al., 2016) using the MagAttract® HMW DNA kit based on the manufacturer protocols (Qiagen GmbH, Hilden, Germany). Briefly, a 1 ml volume of approximately 2 × 10^9^ CFU/ml of freshly overnight cultured bacteria was spanned in 2 ml Eppendorf tubes followed by suspending the pellets in 180 μl buffer ATL (tissue lysis buffer, Qiagen GmbH, Hilden, Germany). Next, Proteinase K was added to each vial at a concentration of 20 mg/ml followed by incubation at 56°C in an Eppendorf thermomixer for 30 min. Afterward, 4 μl RNase was added and the vials were pulse vortexed followed by adding 15 μl of MagAttract Suspension G and 280 μl Buffer MB to each vial (Tarumoto et al., 2017). The suspension from each tube was transferred onto the MagAttract holder followed by mixing for 1 min on an Eppendorf thermomixer. The magnetic beads having the gDNA were separated on the MagAttract magnetic rack for approximately 1 min. Supernatants were removed without disturbing the beads followed by washing the magnetic beads twice using MW1 and PE buffer (Becker et al., 2016; Tarumoto et al., 2017). The remaining suspension was removed by washing the beads twice using 1 ml RNAase free water (Qiagen GmbH, Hilden, Germany) (Becker et al., 2016). The gDNA was harvested by eluting in 100 μl buffer EB while purity was evaluated using the NanoDrop (Thermo Fisher, United States) and gel electrophoresis using 1% agarose. Quantification of gDNA was carried out using the Qubit double-stranded DNA high-CHS kit following the manufacturer’s guidelines (Life Technologies Inc., Carlsbad, CA, United States) (Guan et al., 2020).

### 2.3. Library preparation, sequencing, and bioinformatics analysis

Library preparation was carried out using Nextera DNA Flex (Tagmentation Illumina Inc. San Diego, CA, United States) while Illumina MiSeq were used with a paired-end read length of 2 × 300 bp. The bacterial raw DNA reads were analyzed using the online Galaxy platform^1^ version 21.05. Quality of both forward and reverse raw reads were analyzed using the FastQC Version 0.11.9 software (Bioinformatics B, 2011), while the Trimmometric version 0.38.1 was used to remove the adapters and low-quality reads from paired-end sequences (Bolger et al., 2014). The resulting paired-end sequence reads were *de novo* assembled using SPAdes v. 3.12.0 (Coil et al., 2015) with 33 to 91 k-mers (Bankevich et al., 2012) while genome annotation was done using the prokaryotic genome annotation pipeline (PGAP) (Tatusova et al., 2016) from the National Center for Biotechnology and Information (NCBI) and Prokka (Seemann, 2014). Online Galaxy platform (see Footnote 1) version 21.05 was used for bioinformatic analysis.

### 2.4. Pangenome analysis

Pangenome analysis of *A. veronii* AhS5-24 together with 30 complete genomes of other *A. veronii* isolates retrieved from the NCBI was carried out using Roary v. 3.13.0 using general feature files 3 (.gff) file generated from Prokka v. 1.14.5. The phylogenetic tree was made using the Phandango software using Gene_presence_absence and Newick files obtained from Roary v. 3.13.0. The average nucleotide identity (ANI) of all 31 *A. veronii* genomes was computed using FastANI v1.3 using *A. veronii* FC951 (CP032839) as a reference strain. Antimicrobial resistance (AMR) genes were identified using staramr version 0.7.2 (Tran et al., 2021) and ABRicate v1.0.1 (Seemann, 2016) in the Comprehensive antimicrobial resistance database (CARD) (Alcock et al., 2020) and staramr v. 0.7.2 with the identification threshold set at 80%. Plasmidfinder v 2.0 (Ullah et al., 2020) was used to identify plasmids in the bacterial genomes while virulence genes were identified using virulence factors database (VFDB). Genome circular maps were created using Proksee.^3^

### 2.5. Phylogenetic analysis of antimicrobial resistance genes

Phylogenetic comparison of the *tet(E)* and *tetR* genes from strain AhS5-24 with other *A. veronii* isolates was done using the Molecular Evolutionary Genetic Analysis version 7 (MEGA-7) software (Kumar et al., 2016). The *tet(E)* and *tetR* sequences from strain AhS5-24 were retrieved after screening using ABRicate version 1.0.1 followed by comparison with *tet(E)* and *tetR* sequences from other *A. veronii* isolates retrieved from NCBI. Phylogenetic trees were produced using the Neighbor-joining and BioNJ algorithm to a pairwise matrix estimated using JTT model and expressed as number of base substitution per site (Jones et al., 1992).

## 3. Results

### 3.1. Genome organization and pangenome analysis

**Figure 1:**
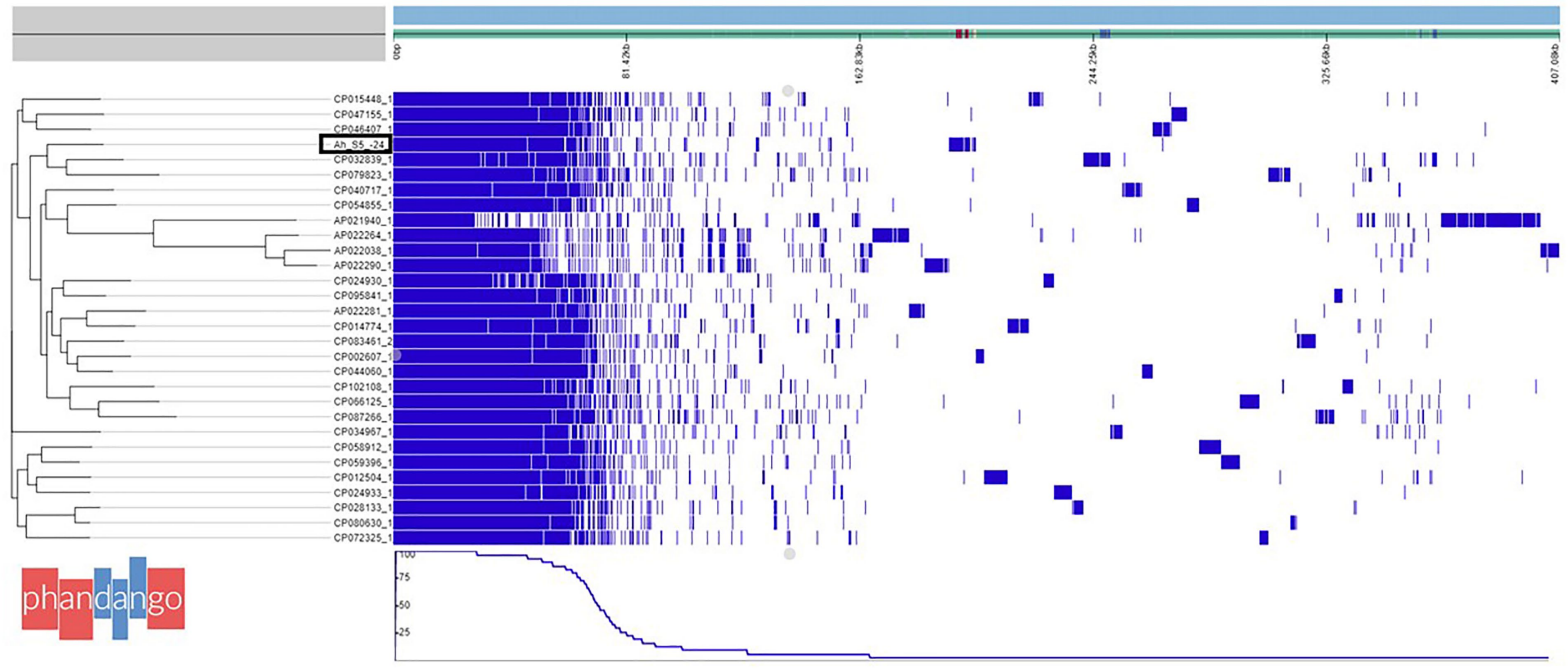
Phylogenetic tree based on pangenome analysis of *Aeromonas veronii* AhS5-24 together with 30 complete genomes of other *A. veronii* strains obtained from the National Center for Biotechnology and Information (NCBI). Note that *A. veronii* AhS5-24 is clustered together with *A. veronii* strains FC951 (CP032839) isolated from hospital sewage. A total of 20352 genes were detected in all 31 *A. veronii* genomes by pangenome analysis using the Roary software of which 1,429 genes were core genes, 875 soft core genes while the number of shell and cloud genes was 2,241 and 15,807 genes, respectively.

The draft genome of *A. veronii* AhS5-24 showed a high similarity with other *A. veronii* genomes, as shown in Figure 1. The draft genome of strain AhS5-24 had a size of 4,748,224 bp with G + C content of 58.48%. It contained 157 contigs with an N50 value of 115,408. A total of 4,493 genes were predicted with 4,334 genes coding for proteins. The genome contained a total of 108 genes of RNA consisting of 99 tRNA and 5 rRNAs. The total number of genes detected from the 31 *A. veronii* genomes based on pangenome analysis was 20,352 of which 1,429 genes were core-, 875 softcore-, 2,241 shell-, and 15,807 cloud genes. The phylogenetic tree divided the genomes into three groups of which strain AhS5-24 was closely related to the human CP032839 (FC951) and hospital sewage CP079823 (HD6454) isolates (Figure 1). The average nucleotide identity (ANI) analysis using FastANI showed high similarity (>93%) of all 31 *A. veronii* isolates despite coming from different host species and geographic locations. The ANI of strain AhS5-24 was 96.31% similar with the *A. veronii* CF951 (CP032839) human clinical isolate and 96.20% similar with *A. veronii* HD6454 (CP079823) from hospital sewage.

**Figure 2:**
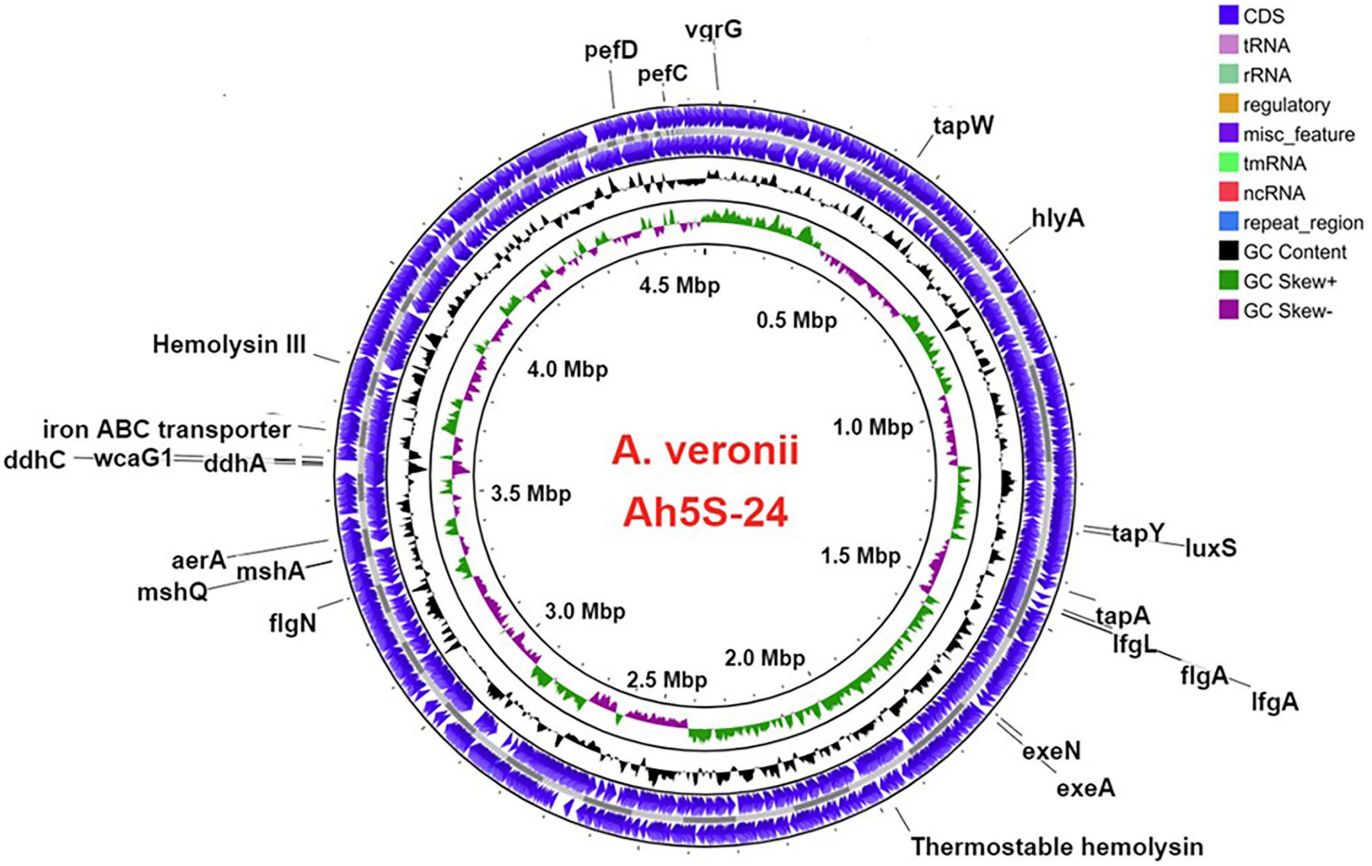
Circular map showing the loci for different virulence genes in the genome of *Aeromonas veronii* strain AhS5-24. Motility and adherence genes detected included the lateral flagella *ifgA* and *IfgL* genes; polar flagella *flaA* and *flgN* genes; tap type IV pili *tapA* and *tapY*; fimbriae *pefC* and *pefD* genes. The mannose-sensitive hemagglutinin (MHSA) genes were represented by *mshA* and *mshQ* while the hemolysin genes included *hylA*, *hylIII,* and *TSH*. Iron acquisition was represented by the iron ABC transporter protein and genes associated with capsule formation included the *ddhA*, *ddhC,* and *wcaG1* genes. Quorum sensing and biofilm formation genes were represented by *luxS*. The T2SS was represented by *exeA* and *exeN* genes while T6SS was represented by the *vgrG* gene.

The virulence genes found in *A. veronii* AhS5-24 comprised the motility and adherence genes that included the (i) lateral flagella proteins consisting of *lfgA* and *lfgL*, (ii) polar flagella that were represented by *flgA* and *flgN*, (iii) members of the tap type IV pili that included *tapA*, *tapW* and *tapY,* and (iv) fimbrial adherence determinants that included *pefC* and *pefD* genes (Figure 2). The mannose-sensitive hemagglutinin (MHSA) is encoded by the genes *mshA* and *mshQ* (Figure 2). Genes associated with capsule formation and immune evasion included *ddhA*, *ddhC,* and *wcaG1*. The hemolysin genes detected were *hlyA*, *hylIII*, and thermostable hemolysin (*TSH*) while toxin genes consisted of aerolysin *aerA*. Genes associated with iron acquisition consisted of the Iron *ABC* transporter while biofilm formation and quorum sensing genes were represented by *luxS* and *MshA*-*Q* pilus. We detected genes belonging to the type II secretion systems (T2SS) represented by *exeA* to *exeN* (Supplementary Table S1) and *vgrG*, which is part of T6SS (Figure 2). Overall, the virulence genes detected belonged to motility, adherence, secretion systems, iron acquisition, biofilm formation, quorum sensing, and immune evasion groups.

### 3.2. Phenotype characterization of antimicrobial resistance genes

Results of the disk diffusion test showed that strain Ah5S-24 was resistant to CFO30, CEP30, AMOXY30, and TET30, whereas it showed intermediate resistance against SULFA240 and TRIM5 (Table 1). However, it was susceptible to ERY15, CO150, CIPR5, GEN10, NI300, and RIF5. We found an overall correlation kappa score of 82% (Cohen’s *k* = 0.8235) with a specificity of 91.66% and sensitivity of 93% between the phenotypic profile based on the disk diffusion test and genotypic profile based on the genes identified using the CARD (Alcock et al., 2020).

### 3.3. Genotype characterization of antimicrobial resistance genes

**Figure 3:**
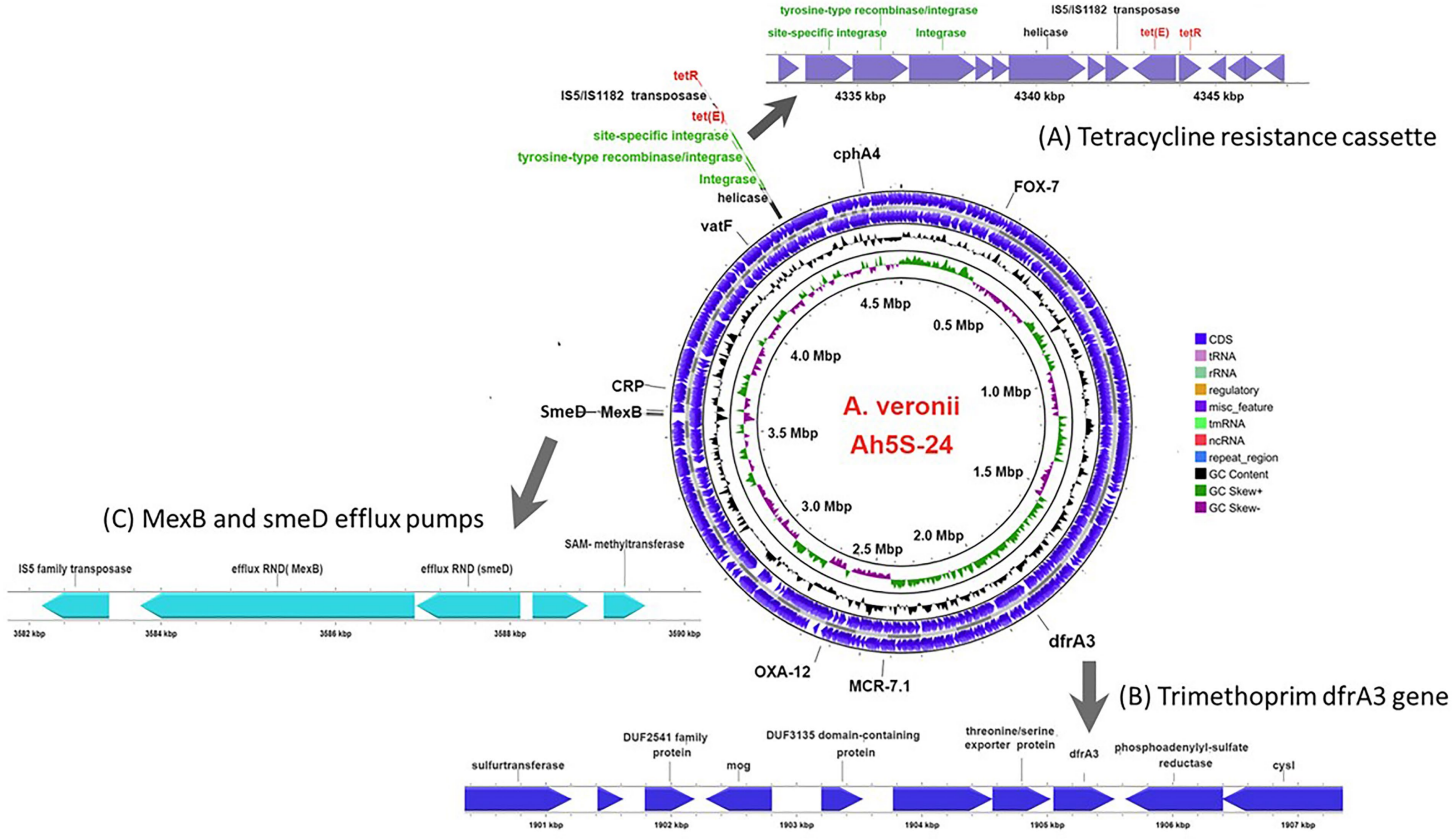
Circular genomic map of *Aeromonas veronii* AhS5-24 showing the loci for antimicrobial resistance (AMR) genes. The AMR genes detected included the *β*-lactam *bla*_OXA-12_ and *bla*_FOX-7_ genes together with *cphA4*, *dfrA3*, *mcr-7.1,* and *vatF* genes while the efflux pump proteins detected were *CRP*, *smedD,* and *mexB*. The extended linear map **(A)** shows the cassette encoding the site-specific integrase, tyrosine-type recombinase/integrase, integrase, helicase, IS*5/*IS*1182* transposase, *tet(E)* efflux pump protein gene and *tetR* gene designated as IS*5/*IS*1182/hp/tet(E)/tetR/hp*. The extended line map **(B)** shows the linear relationship between the trimethoprim *dfrA3* gene and other genes that includes sulfurtransferase, DUF2541 family protein, mog, DUF3135 domain-containing protein, threonine/serine exporter protein, *dfrA3* and phosphoadenyl-sulfate reductase in the genome of *A. veronii* AhS5-24. The extended map **(C)** shows the linear relationship between the *smeD* and *mexB* efflux pumps in the genome of *A. veronii* AhS5-24.

Identification of AMR genes using the CARD (Alcock et al., 2020) showed that strain Ah5S-24 encoded multiple AMR genes that included the *β*-lactamase like *bla*_FOX-7_, *bla*_OXA-12_, and *cphA4*. Other genes detected included the colistin *crp* and *mcr-7.1* genes as well as the streptogramin A acetyl transferase *vatF* gene (Figure 3). There were no integrase and transposases located near the *bla*_FOX-7_, *bla*_OXA-12,_ *cphA4, crp*, *mcr-7.1,* and *vatF* genes. The trimethoprim *dfrA3* gene was placed together with the sulfurtransferase, DUF2541 family protein, mog, DUF3135 domain-containing protein, threonine exporter protein, and phosphoadenyl-sulfate reductase (Figure 3). The efflux pumps detected included the resistance-nodulation-cell division (RND) *mexB* and *smeD* that were placed next to each other together with the IS*5* transposase (Figure 3).

**Figure 4:**
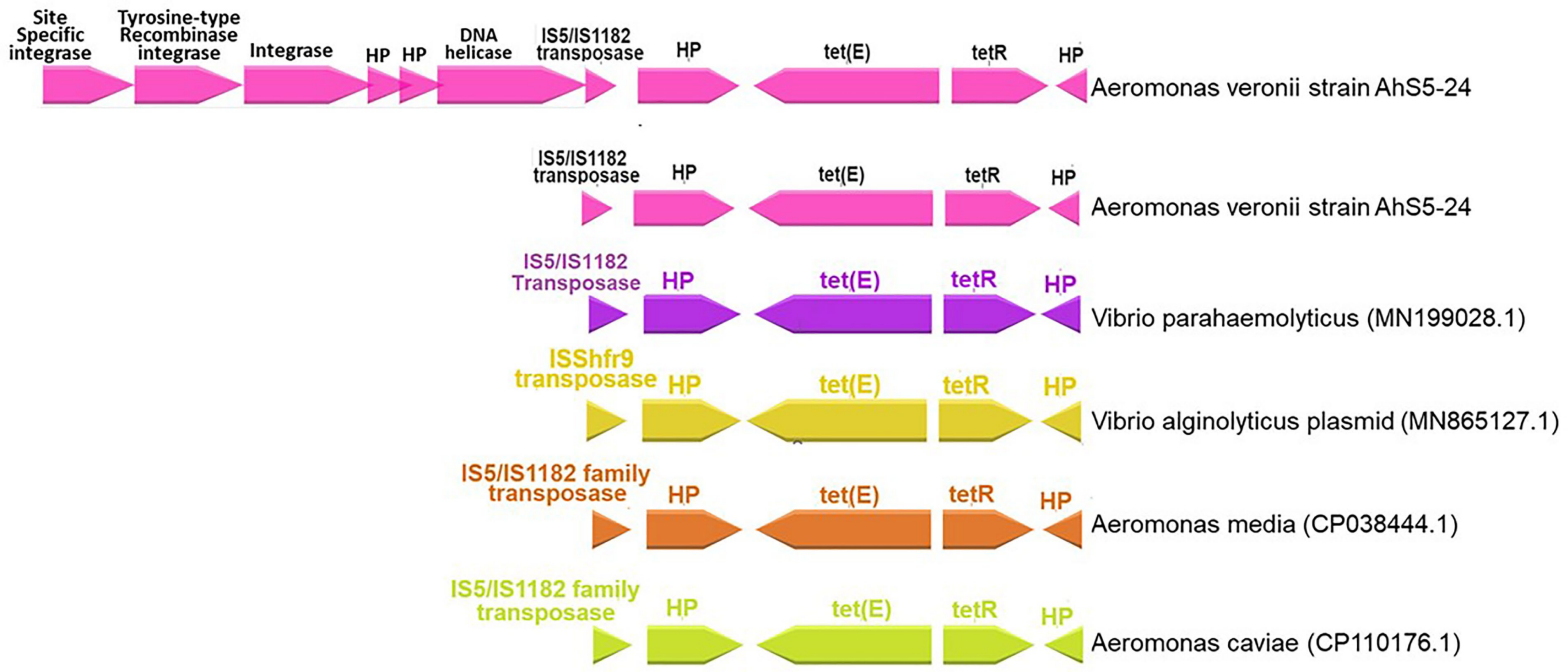
Comparison of the IS*5/*IS*1182/hp/tet(E)/tetR/hp* gene cassettes for *Aeromonas veronii* strain AhS5-24 from pond sediments, *Vibrio parahaemolyticus* (MN199028) isolated from retail fish from a market, *Vibrio alginolyticus* plasmid (MN865127.1) from shrimp, *Aeromonas media* (CP038444.1) from sewage bioreactor and *Aeromonas caviae* (CP110176.1) from human stool. Note that all isolates had the hypothetical proteins (*hp*), *tetR*, *tet(E),* and IS*5/*IS*1182* transposase forming a gene cassette designated as IS*5/*IS*1182/hp/tet(E)/tetR/hp*. The uppermost linear map shows *Aeromonas veronii* strain AhS5-24 having the IS*5/*IS*1182/hp/tet(E)/tetR/hp* cassette linked to the DNA helicase, two hypothetical proteins, integrase, tyrosine-type recombinase/integrase and site-specific integrase.

Our findings show that the repressor of the tetracycline resistance element gene *tetR* was placed next to *tet(E)* together with the IS*5/*IS*1182* transposase, helicase, integrase, tyrosine type recombinase/integrase, and the site-specific integrase all in one cassette (Figure 3). The cassette found in *A. veronii* AhS5-24 showed a high similarity with cassettes found in *Vibrio parahaemolyticus* (MN199028.1) isolated from a fish market, *Vibrio alginolyticus* plasmid (MN865127.1) from shrimp, *Aeromonas caviae* (CP110176) from human stool, and *Aeromonas media* (CP03844.1) from a sewage bioreactor (Figure 4). They all had a similar genetic structure or transposon consisting of the IS*5/*IS*1182* transposase followed by a gene encoding a hypothetical protein (*hp*), *Tet(E)*, *tetR,* and another hypothetical protein (*hp*), thereby forming a MGC designated as 1S*5/*IS*1182/hp/tet(E)/tetR/hp* (Figure 4). Suffice to point out that the cassette from *Vibrio alginolyticus* (MN865127.1) was from a plasmid, while the cassettes from *A. veronii* AhS5-24, *Vibrio parahaemolyticus* (MN199028.1), *A. media* (CP038444.1), and *Aeromonas caviae* (CP110176) were from chromosomes. This findings demonstrate that the IS*5/*IS*1182/hp/tet(E)/tetR/hp* cassette can be found both in chromosomes and plasmids of different bacteria species. It is noteworthy that the cassette for *Vibrio alginolyticus* plasmid (MN865127.1) had the *IShfr9* transposase, and not the IS*5/*IS*1182* transposase, despite having a similar *hp/tet(E)/tetR/hp* component with other bacteria species used in the comparison (Figure 4).

**Figure 5:**
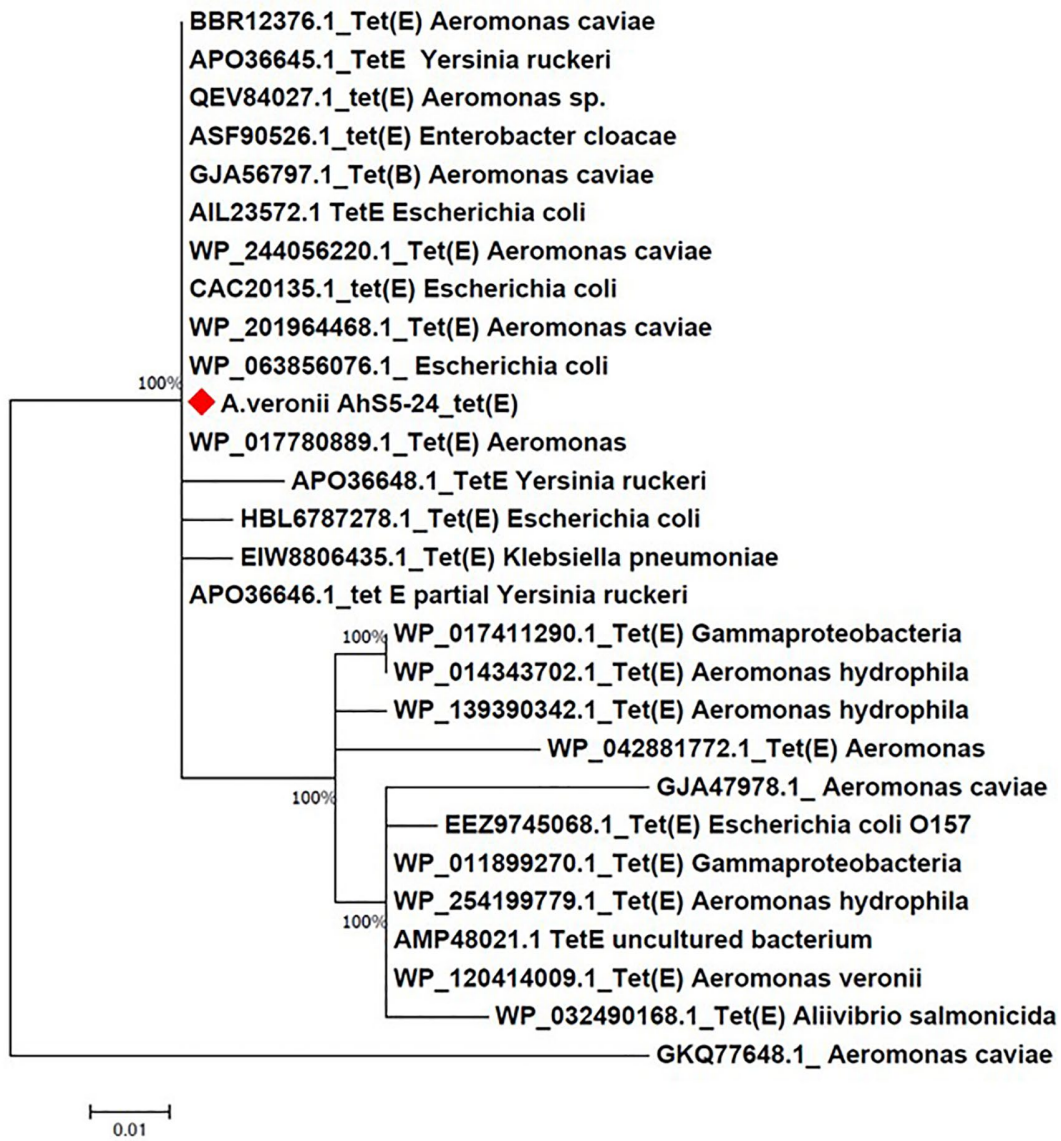
Phylogenetic tree comparing the *tet(E)* gene from different bacteria species. Note that the *tet(E) Aeromonas veronii* strain AhS5-24 had 100% similarity with *tet(E)* genes from other bacteria species that include *Aeromonas caviae* (BBR12376.1, GJA56797.1, and WP/244056220.1 and WP_20964468.1), *Yersinia ruckeri* (Apo036645.1, APO36646.1, and APO36648.1), *Enterobacter cloacae* (ASF90526.1), *Escherichia coli* (AIL23572.1, CAC20135.1, WP_063856076.1 and HBL6787278.1), and *Klebsiella pneumoniae* (EIW8806435.1).

**Figure 6:**
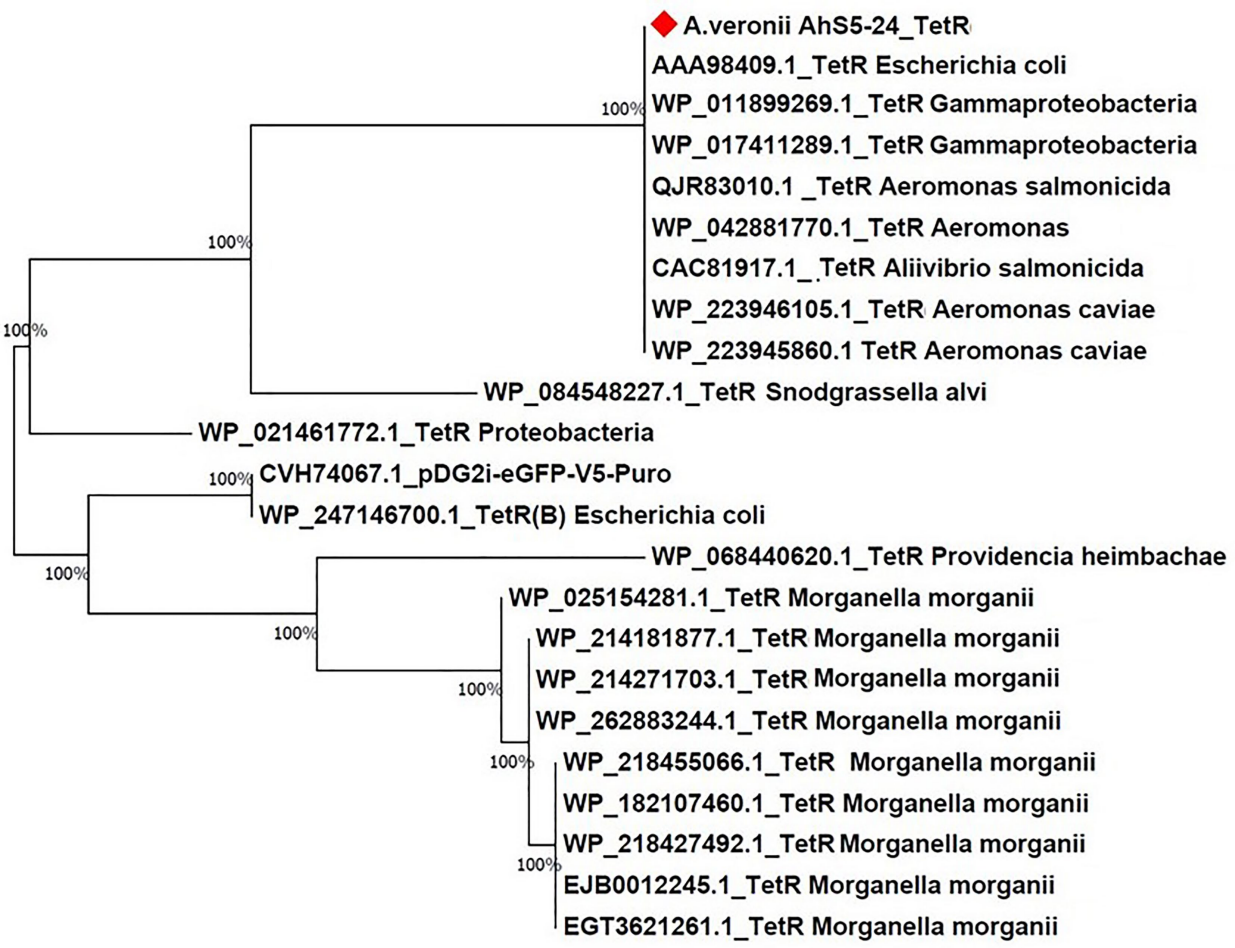
Phylogenetic tree comparing the *tetR* gene from different bacteria species. Note the high (100%) similarity between the *tetR* gene of *Aeromonas veronii* strain AhS5-24 with *tetR* genes of *Escherichia coli* (AAA98409.1), Gammaproteobacteria (WP_011899269.1 and WP_017411289), *Aeromonas salmonicida* (QJR83010.1), *Aeromonas* spp. (WP_04288770.1), and *Aeromonas caviae* (WP_223946105.1 and WP_223945860.1).

Phylogenetic analysis showed that the *tet(E)* gene from *A. veronii* AhS5-24 had a 100% similarity with *tet(E)* genes from different bacteria species that included *Escherichia coli* (AIL23572.1, CAC20135.1, and WP_20194468.1), *Aeromonas caviae* (BBR12376.1, WP_244056220.1, and WP_201964468.1), *Yersinia ruckeri* (APO36645.1, APO36648.1, and APO36646.1), *Klebsiella pneumoniae* (EIW8806435.1), *Aeromonas* spp. (QEV84027.1 and WP_017780889.1), and *Enterobacter cloacae* (ASF90526.1) (Figure 5). Phylogenetic analysis also showed that the *tetR* gene from *A. veronii* AhS5-24 had a 100% similarity with *tetR* genes from different bacteria species that included *E. coli* (AAA98409.1), Gammaproteobacteria (W_P011899269.1 and WP_017411289.1), *Aeromonas salmonicida* (QJR83010.1), *Aliivibrio salmonicida* (CAC81917.1), and *A. caviae* (WP_223946105.1 and WP_223945860.1) (Figure 6). Altogether, our findings show that *tet(E)* and *tetR* genes were highly similar with those found in different bacteria species.

## 4. Discussion

In this study, we have shown that the bacteria isolated from pond sediments in the South East USA previously classified as *A. hydrophila* using the API 20E system in DePaola et al. (1988) was characterized as *A. veronii* Ah5S-24 using WGS and pangenome analysis. We have also shown that the T^r^ and TO^r^ detected by DePaola et al. (1988) could be linked to the *tetR* and *tet(E)* genes found in the same isolate designated as strain Ah5S-24 in this study. In addition, strain Ah5S-24 encoded several virulence and AMR genes of which tetracycline resistance genes were placed in the same genetic structure with an integrase, transposase and recombinase and can be defined as a transposon. These findings demonstrate that *Aeromonas* spp. isolated from aquatic environments have the potential to transmit AMR genes to other bacteria using transposons carrying different AMR genes.

Pangenome analysis showed a high similarity of strain Ah5S-24 with other *A. veronii* strains linked to different diseases in aquatic organism and humans. For example, strains CP032839.1 and CP046407.1 shown to be closely related with *A. veronii* Ah5S-24 were from human clinical cases (Ragupathi et al., 2020) and diseased rohu (*Labeo rohita*) (Tyagi et al., 2022), respectively. Besides, *A. veronii* Ah5S-24 had several virulence genes linked to adherence, biofilm formation, quorum sensing, immune evasion, toxins and intracellular secretion systems (TSS) found in other pathogenic *A. veronii* strains (Arechaga and Cascales, 2022). Detection of the *Msh* pili, tap type IV pili, lateral-and polar flagellar genes associated with intestinal adherence, colonization, and biofilm formation (Ro, 2006; Hadi et al., 2012) is suggestive that these genes play a vital role in the pathogenicity of strain Ah5S-24. The presence of the *LuxS* and *mshQ* genes is suggestive that strain Ah5S-24 has the capacity for biofilm formation and quorum sensing as seen in other bacteria species (Enos-Berlage et al., 2005; Trappetti et al., 2011) while presence of the iron ABC transporter is suggestive that strain Ah5S-24 uses this protein in acquiring iron from infected hosts (Delepelaire, 2019). Detection of *ddhA*, *ddhC,* and *wcaG1* associated with capsule formation (Mobine, 2008) is suggestive strain Ah5S-24 has the ability to form a capsule as a defense mechanism against host immune responses while presence of *hylA*, *hylII,I,* and *TSH* together with aerolysin *aerA* is suggestive that these genes might be linked to pore formation and intracellular release of enterotoxins by strain Ah5S-24 as seen in other bacteria species (Honda et al., 1992; Baida and Kuzmin, 1996; Abrami et al., 2000; Maté et al., 2014). Besides, several scientists (Wong et al., 1998; Heuzenroeder et al., 1999; Wu et al., 2007; Castilho et al., 2009) have shown that a combination of *hylA(+)* and *aerA(+)* is a major virulence determinant in *Aeromona*s spp. Castilho et al. (2009) found a high prevalence of hemolytic and cytotoxic *Aeromonas* spp. that had both *hylA(+)* and *aerA*^+^ from human clinical, food, and environmental samples in Brazil while Wu et al. (2007) showed that the absence of *hlyA(−)* and *aerA(−)* in *Aeromonas* spp. from fish and human samples in Taiwan was associated with low virulence. Heuzenroeder et al. (1999) and Wong et al. (1998) showed that deletion or attenuation of the *hlyA(+)* and *aerA(+)* double mutant significantly reduced the pathogenicity of *A. hydrophila* in mice. They also showed that cytotoxicity to buffalo green monkey kidney cells and hemolysis on horse blood agar was only eliminated in the double and not in the single mutants of *A. veronii*, *A. hydrophila*, and *A. caviae*. Our findings show that *A. veronii* Ah5S-24 had both *hlyA(+)* and *aerA(+),* indicating that it shares the two key virulence determinants with other pathogenic *Aeromonas* spp.

Several studies have shown that *Aeromonas* spp. intrinsically carry various *bla*_OXA_ genes in their genomes that include the *bla*_OXA-12_ gene (Dubey et al., 2022a,b) previously detected in *A. media*, *A. jandaei*, *A. sobria*, *A. dhakensis,* and *A. hydrophila* (Dubey et al., 2022a; Rasmussen et al., 1994; Alksne and Rasmussen, 1997; Hilt et al., 2020; Huang et al., 2020) being in line with its presence in strain Ah5S-24 while *bla*_FOX-7_ previously reported in *A. media* and *A. allosaccharophila* was also found in strain Ah5S-24 (Ebmeyer et al., 2019). Other AMR genes detected included the *cphA4* gene known to be intrinsically encoded in various *Aeromonas* spp. (Dubey et al., 2022a,b) as well as the colistin-resistance *mcr-7.1* gene also reported from different *Aeromonas* spp. (Dubey et al., 2022a,b). Despite so, the *bla*_OXA-12_, *bla*_FOX-7_, and *mcr-7.1* genes detected in strain Ah5S-24 were not associated with integrases, recombinases or transposases suggesting that these genes could not be easily transferred or acquired from other bacteria species. Similarly, although trimethoprim and sulfonamide are among the most widely used antibiotics linked to AMR in aquaculture (Gao et al., 2012; Muziasari et al., 2014; Phu et al., 2015), the trimethoprim resistance gene *dfrA3* detected in the present study was not linked to integrases and transposases. Thus, the sulfonamide and trimethoprim resistance observed in the disc diffusion test could have been mediated by the *MexB* and *smeD* pumps that have been associated with resistance of several drugs that include sulfonamide, fluoroquinolone, cephalosporins, carbapenem, and trimethoprim. The trimethoprim and sulfonamide resistance observed on the disc diffusion test was intermediate (I) unlike the tetracycline resistance (R), which was highly expressed suggesting that the impact of trimethoprim and sulfonamide in conferring resistance was not as high as tetracycline in strain AhS5-24. Despite so, we found a high correlation of kappa score of 82% (Cohen’s *k* = 0.8235) with a specificity of 91.66% and sensitivity of 93% between the phenotype characterization based on the disc diffusion test and genotypic characterization based on the CARD (Alcock et al., 2020), indicating that the two diagnostic tests were highly in agreement.

Tetracycline is one of the most widely used antibiotics in aquaculture, which has been linked to resistance in farmed aquatic organisms (Seyfried et al., 2010; Tamminen et al., 2011). Thus, it is likely that selection of the Tet E operon in strain AhS5-24 occurred in pond sediments used for aquaculture where tetracycline was used for the treatment of fish diseases. Although the absence of plasmids is suggestive that strain Ah5S-24 had lesser chances of transferring AMR genes to other bacteria, detection of the Tet E operon together with the integrase and IS*5/*IS*1182* transposase suggests that *tetR* and *tet(E)* genes could be transferred or acquired from other bacteria using the IS*5/*IS*1182/hp/tet(E)/tetR/hp* cassette encoded in strain Ah5S-24. Besides, DePaola et al. (1988) used the same isolate to transfer the OT^r^ and T^r^ resistance to *E. coli* suggesting that the IS*5/*IS*1182/hp/tet(E)/tetR/hp* cassette found in strain AhS5-24 could have been responsible for transferring the tetracycline resistance to *E. coli*. Also, detection of the same cassette in *V. parahaemolyticus*, *V. alginolyticus* (MN199028.1), *A. media* (CP038444.1), *A. caviae* (CP110176.1), and *A. caviae* (CP038445.1) emanating from fish market, shrimp, sewage bioreactor and human stool is suggesting that the IS*5/*IS*1182/hp/tet(E)/tetR/hp* transposon could be involved in interspecies transmission of the *tet(E)* and *tetR* genes in different bacteria species. These findings also suggest that the IS*5/*IS*1182/hp/tet(E)/tetR/hp* transposon might be in existence in different bacteria species found in different aquatic environments hosted by species that include shrimps, fish, animals and humans. Its presence in *V. parahaemolyticus* (MN199028.1) isolated from retail fish at markets and *V. alginolyticus* (MN865127.1) from shrimps is indicative that it could play a vital role in transmission of *tet(E)* and *tetR* genes to humans through food.

The similarity of the IS*5/*IS*1182/hp/tet(E)/tetR/hp* cassette found in the chromosomes of strain *A. veronii* AhS5-24, *V. parahaemolyticus* (MN199028.1) and *A. media* (CP038445.1), with the transposon found in the plasmid of *V. alginolyticus* (MN865127.1) is suggesting that the IS*5/*IS*1182/hp/tet(E)/tetR/hp* transposon can be transferable between chromosomes and plasmids of different bacteria species. Also, the high similarity of the *tet(E)* and *tetR* genes detected in strain AhS5-24 with those found in *E. coli*, *K. pneumoniae,* and *Aeromonas* spp. shown in the phylogenetic analysis consolidates our view that *tet(E)* and *tetR* genes could be transmissible between different bacteria species using MGCs. Thus, it is likely that the transfer of the OT^r^ and T^r^ resistance to *E. coli* observed by DePaola et al. (1988) was not plasmid mediated but it was done by the IS*5/*IS*1182/hp/tet(E)/tetR/hp* transposon found in strain Ah5S-24. Therefore, our findings indicate that the resistance acquired by different *Aeromonas* spp. in aquatic environments could play a vital role in the transfer of AMR genes to foodborne, environmental, nosocomial and other bacteria species using MGCs. However, future studies should seek to demonstrate the transfer of *tet(E)* and *tetR* genes using the IS*5/*IS*1182/hp/tet(E)/tetR/hp* cassette to other bacteria spp. including nosocomial, foodborne and environmental bacteria.

## 5. Conclusion

In this study, we have shown that *A. veronii* AhS5-24 is a multidrug-resistant bacterium encoding several AMR and virulence genes. It encoded a tetracycline resistance operon Tet E placed in a transposon designated as IS*5/*IS*1182/hp/tet(E)/tetR/hp* found in different bacteria species inhabiting different aquatic environments and infecting different host species suggesting that the Tet E operon could be transferred to other bacteria. Overall, this study shows that MGCs encoding AMR genes found in bacteria inhabiting aquatic environments could play a vital role in the spread of AMR genes to other bacteria infecting animals and humans.

## Data availability statement

The *Aeromonas veronii* whole genome shotgun (WGS) project has the project accession JAJVCX000000000. This version of the project (01) has the accession number JAJVCX010000000 and consists of sequences JAJVCX010000001-JAJVCX010000157.

## Author contributions

SD, HS, and HM: conceptualization, methodology, mobilizing resources, supervision, data curation, and bioinformatics analysis. SD, EA-W, BP, AD, HS, and HM: manuscript preparation, editing, and submission. All authors contributed to the article and approved the submitted version.

## Funding

This study was financed by the Research Council of Norway (FIFOSA-21 Project) Grant Number 320692 and the National Natural Science Foundation of China (NSFC) Grant Number 32061133007.

## Conflict of interest

AD was employed by Angelo DePaola Consulting LLC.

The remaining authors declare that the research was conducted in the absence of any commercial or financial relationships that could be construed as a potential conflict of interest.

## Publisher’s note

All claims expressed in this article are solely those of the authors and do not necessarily represent those of their affiliated organizations, or those of the publisher, the editors and the reviewers. Any product that may be evaluated in this article, or claim that may be made by its manufacturer, is not guaranteed or endorsed by the publisher.

## Footnotes

^1^https://usegalaxy.no/

^3^https://proksee.ca/
